# Distribution of Two Strains of *Leptoglossus zonatus* (Dallas) (Hemiptera: Coreidae) in the Western Hemisphere: Is *L. zonatus* a Potential Invasive Species in California?

**DOI:** 10.3390/insects12121094

**Published:** 2021-12-07

**Authors:** Andrea L. Joyce, Hannah Parolini, Harry Brailovsky

**Affiliations:** 1 Department of Public Health, University of California Merced, 5200 N. Lake Road, Merced, CA 94343, USA; hparolini@ucmerced.edu; 2 Instituto de Biología, National Autonomous University of Mexico (UNAM), Mexico City 04510, Mexico; coreidae@ib.unam.mx

**Keywords:** range expansion, strains, haplotype, invasive species, Coreidae, leaffooted plant bug

## Abstract

**Simple Summary:**

The leaffooted plant bug, *Leptoglossus zonatus*, is widely distributed in the Western Hemisphere. In California, two lineages (strains) occur. One lineage is known from California, and the second is found in California and Brazil. Although this species has been in California since 1900, it has become a pest in almonds in the last decade. It is possible that a cryptic species or strain has been introduced. This study investigated the distribution of the two lineages (strains) of *L. zonatus* in the Western Hemisphere. Specimens from the *Leptoglossus* collection in the national insect collection in Mexico were used to extract DNA and sequence the mitochondrial DNA cytochrome oxidase I gene. These sequences were combined with others in Genbank from California and South America to determine the strain distributions. The first strain occurred in California and Mexico, while the second was widespread from California into South America. When all samples were combined, there was overall low genetic diversity. The small number of genetic types (haplotypes), the range expansion, and the economic pest status of *L. zonatus* in California, suggest this insect is a potentially invasive insect pest.

**Abstract:**

The leaffooted plant bug, *Leptoglossus zonatus* (Dallas) (Hemiptera: Coreidae) is polyphagous and widely distributed in the Western Hemisphere. Although it has been recorded in California since around 1900, it has become a more common pest in almonds in the last decade. Other studies have shown that an established insect can become a pest when a new genotype is introduced. This study investigated the distribution of two lineages (strains) of *L. zonatus* in the Western Hemisphere. Specimens from the *Leptoglossus* collection in the national insect collection in Mexico were used to extract DNA and sequence the mitochondrial DNA cytochrome oxidase I (mtDNA COI) gene, for use in population genetic and phylogenetic analyses. New sequences from Mexico, Central and South America were combined with those available in GenBank, from California and Brazil. Two lineages (strains) of *L. zonatus* were uncovered. One lineage occurs in California, Mexico and Ecuador. The second lineage is more widespread and found in California, Mexico, Guatemala, Nicaragua, Bolivia and Brazil. The haplotype number and diversity, and nucleotide diversity, were found for samples from California, Mexico, and Brazil, for the two lineages, and for all 118 sequences combined. All sequences combined produced five haplotypes, and a haplotype diversity of 0.54. California and Brazil had 3 haplotypes each, with one haplotype shared (5 total). Haplotype diversity in California and in Brazil were 0.526 and 0.505, respectively. A haplotype network found that one haplotype was most abundant and widespread. The small number of haplotypes, a range expansion, and economic pest status of *L. zonatus* in California, all contribute to this insect being a potentially invasive insect pest.

## 1. Introduction

*Leptoglossus* species (Guérin-Méneville) (Hemiptera: Coreidae), also known as leaffooted plant bugs, are endemic to the Western Hemisphere with at least 61 species recorded [1]. The species *Leptoglossus zonatus* (Dallas) has a broad distribution from the southern portion of North America, through Central America, and into north and central South America [2]. This species is recorded from dozens of host plant species including many which are economically important such as maize, citrus, tomato, cotton, pistachio and almonds [3,4,5,6,7,8,9,10,11]. In the last decade, *L. zonatus* has increased in abundance in California on almonds [12,13]. The apparent increase of *L. zonatus* could be attributed to factors including the increase in almond acreage in California [14,15], a changing climate which alters insect development and reproduction [16], or through the introduction of cryptic species or strains which are morphologically similar yet vary in their biological attributes [17].

Earlier accounts of *L. zonatus* in North America reported this species from the southern region of California, and in Arizona and Texas [2,18]. This species was introduced into Louisiana in 1996 and was subsequently detected in Florida in 2005 [19,20]. In 2013, *L. zonatus* was reported in the northern part of the Central Valley of California [10]. *L. zonatus* occurs in 13 countries, and of the at least 61 reported *Leptoglossus* species, is the most widely distributed in the Western Hemisphere [1,21]. The native region where this species has more genetic variability is currently unknown.

In the Central Valley of California, *L. zonatus* consists of two genetically divergent populations and lineages. In the Hemiptera, the genetic distance between two species typically ranges up to 5% [22]. The two *L. zonatus* lineages in California are morphologically similar and approximately 2% genetically divergent, therefore considered the same biological species. Biological parameters such as generation time, fecundity, and susceptibility to natural enemies such as parasitoids and predators, and whether or not the two lineages have distinct pheromone blends remains to be investigated [23,24,25,26,27,28].

The two *L. zonatus* lineages in the Central Valley of California vary in their distributions. One strain occurs from the North to South throughout the Central Valley and had not been previously sequenced from outside of California [10]. A second strain of *L. zonatus* was collected in the southern region of the valley; individuals of the second strain were genetically identical to sequences available of *L. zonatus* from Brazil [10]. The second strain which is found in both California and Brazil could be widespread with a distribution throughout the Western Hemisphere, or if it may be a genotype recently introduced into California. Other insects such as the sugarcane aphid have been established, but introduction of a new genotype can elevate the insect to a new pest or invasive status [29,30]. Higher haplotype diversity or genetic diversity in one region could suggest the region of origin, while an area with a lower genetic diversity could indicate a population or genotype has been introduced outside of its native range [31,32,33].

To further characterize the distribution of the two *L. zonatus* strains, *L. zonatus* specimens were sequenced from Mexico, Central and South America. Samples were combined with other available sequences which mostly came from California and Brazil. Haplotype diversity was examined in each region, as well as within each lineage. In addition, sequences were examined for the presence of cryptic species, which are morphologically similar to *L. zonatus*, but sufficiently genetically divergent to be characterized as distinct species. Data were considered as to whether or not this may be an introduced or invasive species in California.

## 2. Methods

### 2.1. Sample Collection, DNA Extraction and Sequencing

Adult *L. zonatus* specimens were obtained from the National Insect Collection at Universidad National Autonoma de Mexico (UNAM) in Mexico City, including samples of *Leptoglossus zonatus* from Mexico and from throughout the Western Hemisphere. In addition, several specimens were included of *L. vexillatus* (a synonym of *L. zonatus*) [2], to determine if the two species were genetically similar. Adult *Leptoglossus* individuals in this study were identified to species based on adult morphological characteristics.

*L. zonatus* samples included came from a variety of collection years from 1948 to 2012 and from locations in Mexico as well as from other Central and South American countries, when samples were available (Table 1, Supplemental Table S1). Two samples of a closely related species, *Leptoglossus neovexillatus* were included as an outgroup (Table 1). A front leg of each of 75 specimens was used for DNA extraction. DNA was extracted using the Qiagen DNeasy Blood and Tissue kit following standard extraction protocols and with an overnight incubation. The DNA was used to sequence a ~600 bp region of mitochondrial DNA cytochrome oxidase 1 (CO1) using two sets of primer pairs; one was the universal forward primer LCO 1490 (5′-GGTCAACAAATCATAAAGATATTGG-3′) and reverse primer HCO 2198 (5′-TAAACTTCAGGGTGACCAAAAAATCA-3′). If the first primer did not produce a sequence, a second primer pair was used; the second was LepF2_t1 5′-ATTCAACCAATCATAAAGATAT-3′ and LepR1_ 5′-TAAACTTCTGGATGTCCAAAAA-3′) [22,25]. A PCR mix consisted of the following: 195.6 µL sterile ultra-pure water, 2.4 µL Taq polymerase (Clonetech, Mountain View, CA, USA), 30 µL Taq 10× buffer, 24 µL dNTPs, 6 µL forward primer, and 6 µL reverse primer; this mix was for six reactions. For each reaction, 5 µL template DNA was added to each vial and the contents were vortexed and spun down. The PCR program was the following: an initial 1 min warm-up at 95 °C; then 40 cycles of 92 °C for 30 s, 43–52 °C for 30 s, and 72 °C for 60 s with a 68 °C final extension for 10 min. PCR products were visualized on a 1.5% agarose gel. Samples were cleaned-up using the Exo-sap-it (Affymetrix, Inc., Santa Clara, CA, USA) cleanup kit and run on a 3730 Genetic Analyzer.

### 2.2. Phylogenetic and Genetic Diversity Analyses

The software Geneious 7 (Biomatters, Aukland, New Zealand) was used to visualize, edit, and align sequences, and to produce consensus sequences [34]. Additional mtDNA COI sequences of *L. zonatus* from Brazil and California available in GenBank were included for comparison [10]. Sequences were aligned in Geneious 7.0 using the Clustal W alignment function and used to produce an unrooted neighbor-joining tree [34]. Bootstrap support values were obtained by 1000 pseudo replicates of the aligned data set, and those above 60% are shown below supported nodes [35]. In addition, a phylogenetic tree was produced using model-based maximum likelihood (ML) analysis for the same dataset. Using the model selection option in MEGA 7.0, we found that the TN92 was the best-fit model to our dataset based on the lowest BIC (Bayesian Information Criterion) value [36]. Pairwise genetic distances among the individual sequences were calculated using Geneious.

Mitochondrial DNA COI sequences produced from this study were used to determine the number of haplotypes, haplotype diversity, and nucleotide diversity using DnaSP 5.10 [37]; these parameters were determined for the datasets from Mexico, and also from datasets from California and Brazil, as well as from the total combined sample dataset. In addition, all *L. zonatus* from strain 1 and strain 2 were separately analyzed to determine the same parameters described above. Results were used to construct a haplotype network using Pop Art 1.7 and a TCS network [38]. All the sequences generated in this study were submitted to GenBank.

## 3. Results

The mtDNA COI sequences were obtained for 35 of the 75 adult *L. zonatus* samples from the collection at UNAM in Mexico (Table 1, Supplemental Table S1). The year of collection of the samples included was from 1957–2012 (Table 1, Supplemental Table S1). The majority of sequences successfully obtained were from museum specimens (31/35) collected from the year 2000 or more recently (Table 1), while sequences were only obtained from several samples collected prior to 2000; one was from 1982, and three were from the 1990s (Table 2). The majority of samples which produced a sequence were from Mexico (Table 1).

### 3.1. Phylogenetic Trees

**Figure 1:**
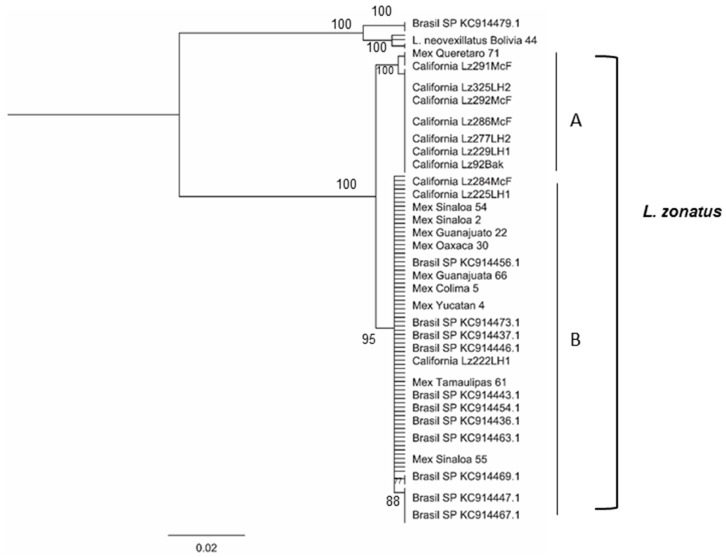
Maximum likelihood tree including *L. zonatus* and *L. neovexillatus* samples. Within the *L. zonatus* samples, (A) indicates lineage one, the top cluster which includes samples from only California and Mexico; (B) is lineage two, the larger cluster on the bottom of the tree, which shows samples from California, Mexico, and Brasil.

Of the 35 sequences produced from the insect collection at UNAM in Mexico, 30 were from Mexico, 3 from Bolivia, 1 from Ecuador, and 1 from Nicaragua. Other sequences included in the tree included 41 samples previously sequenced from the Central Valley of California (Genbank Accessions MF669762-MF669802) [10], and 42 *L. zonatus* COI sequences available in GenBank from the Sao Paulo region of Brazil (Genbank Accessions KC14435-KC14443, KC14445-KC14464, KC14466-KC14471, KC14473-KC14470); these datasets together consisted of 118 sequences. For the Mexico, California and Brazil datasets, a maximum likelihood tree was produced for the combined data set using all 118 sequences (Figure 1).

The combined tree of the 118 samples had two primary lineages well supported (Figure 1). There was a large genetic distance (13%) between the two main lineages, suggesting the presence of a cryptic species which resembles *L. zonatus.* The individuals on the distant lineage are *L. neovexillatus* Allen, an outgroup to *L. zonatus*. This group included two specimens from Bolivia, and five from Brazil which were identified in Genbank as *L. zonatus.*

The second major lineage of the phylogenetic tree consisted of individuals of *L. zonatus*, and was split into two sub-branches with a genetic divergence of ~2% between them (Figure 1). The first sub-branch of *L. zonatus* consisted of California samples, and four specimens from northern and central Mexico (Mex; Queretaro 71,73; Sonora 15; Sinaloa 57). Clustered with California and Mexico *L. zonatus* samples was an individual *L. vexillatus* adult from Ecuador. *L. vexillatus* is a synonym of *L. zonatus*, morphologically identical, and the COI bar code grouped with *L. zonatus* (Figure 1). The second sub-branch of *L. zonatus* individuals were specimens collected throughout Mexico, as well as samples from California, Brazil, Central and South America.

**Figure 2:**
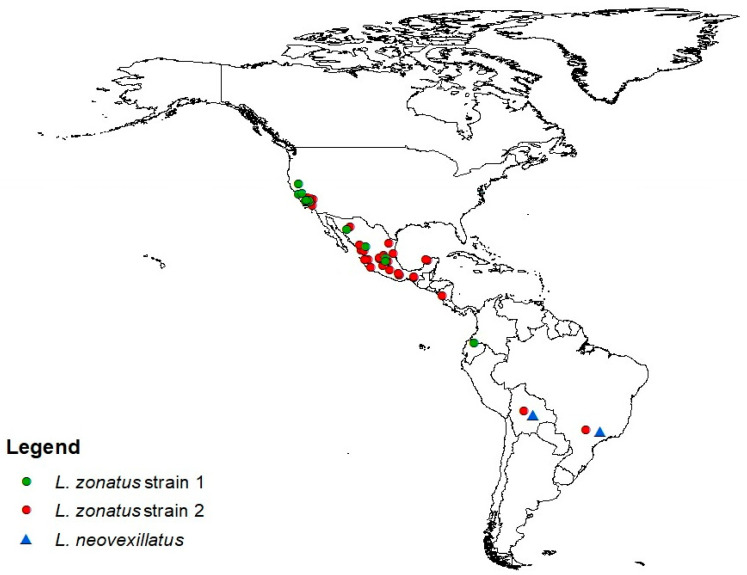
Distribution of *L. zonatus* strain 1 and 2 from specimens in this study and from previously sequenced samples. Circles represent collection locations, not abundance. Blue triangles represent *L. neovexillatus*, a closely related species.

The majority of Genbank sequences of *L. zonatus* from Brazil (37/43) grouped with the second *L. zonatus* sub-branch which consisted of a widespread haplotype; five individuals misidentified as *L. zonatus* in Genbank grouped with *L. neovexillatus,* the highly divergent (13%) lineage. The distribution of the two genetic types of *L. zonatus* obtained in this study, and from previous sequences is mapped (Figure 2).

### 3.2. Haplotype Determination, Haplotype and Nucleotide Diversity

The number of haplotypes, the haplotype diversity, and nucleotide diversity, were determined for mtDNA COI sequences for each data set (Mexico, California, Brazil), and for the combined dataset. In addition, samples which grouped into sub-branch 1 (strain 1) of *L. zonatus*, and those which clustered with sub-branch 2 (strain 2) of *L. zonatus*, were used to determine the same genetic diversity parameters described above.

The *L. zonatus* samples from California consisted of 3 haplotypes. In California, *L. zonatus* strain 1 consisted of 2 haplotypes, with a relatively low haplotype diversity of 0.083; when combined with the samples from Mexico, strain one had a haplotype diversity of 0.198 (Table 3). In California, the third haplotype was from strain 2 (the widespread strain) and all were one haplotype (no genetic diversity) which was genetically identical to the majority of specimens found in Brazil, Mexico, Nicaragua, and Bolivia (Table 3).

The *L. zonatus* from Brazil all grouped into one lineage (sub-branch/strain 2), comprised of 3 haplotypes, and a relatively moderate haplotype diversity of 0.55 (Table 3). When the individuals of the widespread haplotype (with no genetic diversity) were removed from the analysis, the haplotype diversity was reduced to 0.245.

**Figure 3:**
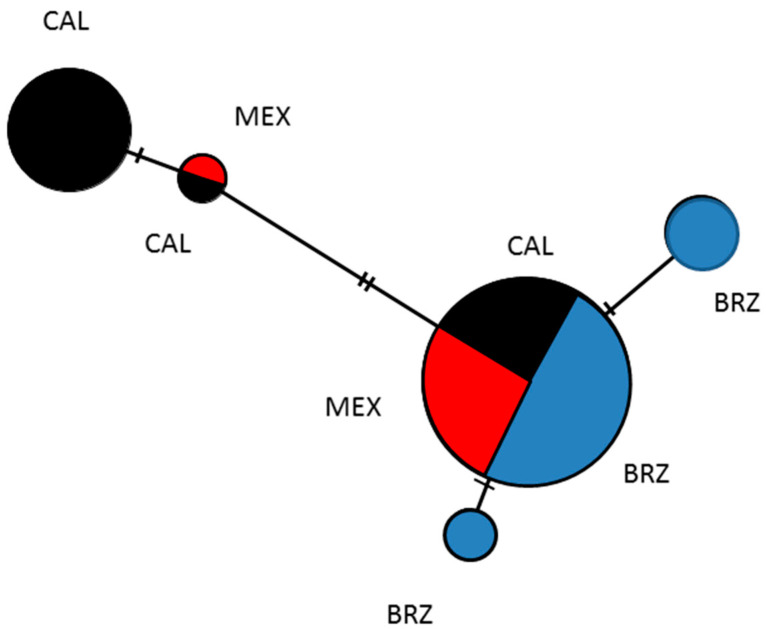
Haplotype tree of the haplotypes of *L. zonatus* sequenced from California (CAL) shown in black, Mexico (MEX) is in red, and Brazil (BRZ) in blue.

When all samples from this study and those previously available were combined they consisted of five haplotypes (Figure 3). Two of the haplotypes were especially abundant, Haplotype 1 in California and Haplotype 3 found from California to Brazil (Figure 3). However, it was the Brazil haplotype which was most abundant, found with a larger distribution and zero genetic diversity.

## 4. Discussion

This study sequenced *L. zonatus* from Mexico, Central America, and South America. Two genetically divergent lineages of *L. zonatus* were detected. One of these lineages was found to occur in California, Mexico, and Ecuador. A second lineage of *L. zonatus* was widespread, ranging from California to Brazil: many of the *L. zonatus* samples in the second lineage were the same haplotype, genetically identical. In total, five haplotypes of *L. zonatus* were found, even when combining samples with previously sequenced *L. zonatus*. The small number of haplotypes suggest that these *L. zonatus* are not likely in their center of origin. Finally, the reported range of *L. zonatus* has expanded from Southern California into Northern California, and also into Louisiana and Florida in North America where it was not previously reported, in the last few decades.

A previous study of *L. zonatus* in California similarly found two genetically distinct lineages. The first lineage occurs from the northern to the southern end of the central valley. There are three haplotypes of the first lineage; sequences are from California, Mexico, and Ecuador, with moderate haplotype and nucleotide diversity. The second lineage in California has a more limited distribution, from the mid to southern half of the Central Valley; however, outside of California it is represented by one haplotype which occurs from California to Brazil. Most of the samples sequenced from Mexico belong to this second lineage. Gene flow was observed between these two lineages in California, evidenced by hybrids of the two genotypes near Delhi, in central California [10].

*L. zonatus* in California is occasionally an abundant pest, and hundreds of insects have been observed mating and aggregating on almonds during the growing season, and at harvest time; in addition, large aggregations of *L. zonatus* overwinter on pomegranate. The occasional high abundance of *L. zonatus* in California is a phenomenon of the last decade, which suggests that a cryptic species or strain may have been introduced. It is possible that one of the *L. zonatus* lineages has expanded its range in California. Earlier accounts of *L. zonatus* report it from Southern California and into Los Angeles and Pasadena, but not from the northern Central Valley. In the last two decades, this species has also been detected in Louisiana and Florida; those populations do not yet have published sequences. The lineage of *L. zonatus* which appears to be more widespread (Brazil, Bolivia, Nicaragua, Mexico, California) may be more adaptable to a variety of climatic conditions found throughout its large range, and could be the lineage or genotype introduced into Florida and Louisiana.

One interesting finding is that the two *L. zonatus* lineages co-occur and were collected in several locations in California and Mexico on the same date. For example, both types were found in California at Lost Hills on two dates (2013 and 2014) and in McFarland, California (2014), and found together in Mexico in Sinaloa (2010) and Queretaro (2007) on the same collection dates. Perhaps the widespread lineage (strain) has a reproductive advantage, such as higher fertility, or more generations per year. While the number of individuals in lineage two is low in some collection sites [Bolivia (1), Nicaragua (1)], in Mexico there were 15 collection sites from 12 sampling years with the widespread lineage predominant. It is possible that if larger sample numbers were available from these collection sites, higher haplotype diversity could have been detected. Another possibility is that the widespread lineage is replacing the more restricted lineage. This would need to be investigated with older museum specimens.

*L. neovexillatus* was included due to its morphologically similar to *L. zonatus*; this species formed another lineage 13% divergent. This group consisted of two *L. neovexillatus* specimens from Bolivia and several GenBank sequences from insects identified as *L. zonatus* from Brazil. *L. neovexillatus* is recorded from Argentina, Bolivia, Brazil, Paraguay, Peru, and Uruguay [2]. This species has a distribution in the southern portion of Brazil with some overlap with *L. zonatus*. The *L. neovexillatus* from Brazil appeared sufficiently morphologically similar enough to be mistakenly grouped with other *L. zonatus* from Brazil, yet genetically those specimens were quite genetically divergent. The *L. zonatus* group could benefit from a study of genetic distance among members to examine evolutionary relationship and to compare with existing taxonomic studies [2,21].

Genetic diversity can help determine where a species may be native, introduced, or invasive. For example, the number of haplotypes for a species can be compared between regions where it is native or suspected to be introduced. There was a low haplotype (5) and moderate genetic diversity for *L. zonatus* in this study. While the precise region of origin and genetic diversity is not known for *L. zonatus*, the number of haplotypes found can be compared to those obtained for other *Leptoglossus* species. *Leptoglossus clypealis* Heidemann in California was genotyped from three sites within 100 km in the Central Valley of California. Among the 20 *L. clypealis* genotyped using mtDNA COI, there were 17 haplotypes with a haplotype diversity of 0.979, suggesting that California is a native region for this species [10,39]. Another related species, *L. occidentalis* Heidemann, has been introduced from North America into Europe. Prior to arriving in Italy, *L. occidentalis* slowly spread from the Western United States through the Midwest and into the Eastern United States [40,41]. Using mtDNA CytB haplotypes, 48 haplotypes were found in W. North America, with 5 haplotypes in Eastern N. America, and 4 haplotypes in the introduced range [33].

When an insect is introduced on another continent, a range expansion can more easily be observed, such as with *Halyomorpha halys* Stal (Hemiptera: Pentatomidae), the brown marmorated stink bug (BMSB). The BMSB is native in China and Japan where more than 25 haplotypes were observed, while only five were observed in introduced areas including in the US [42]; in China and Japan, haplotype diversity was high 0.942 and 0.858. Insects in their region of origin typically demonstrate high haplotype numbers. Four Pentatomidae (Hemiptera) considered native in California were found to have high haplotype numbers and diversity [43], as were a collection of *Lygus hesperus* (Knight) from its endemic region of the Western US [31].

Sample sizes genotyped from California, Mexico and Brazil, were sufficient to detect genetic variability yet low genetic and haplotype diversity was found [10]. Some sample locations in the present study had small sample sizes or one sample was obtained (Nicaragua), which could lead to the overrepresentation of a common haplotype. A larger number of the *Leptoglossus* species occur in South America [1,2,21]. An examination of *L. zonatus* genetic diversity from that region could uncover more haplotypes than found in this study, and potentially find a region where there is higher genetic and haplotype diversity for this species.

*L. zonatus* may have been moved from its center of diversity inadvertently by humans, as is the case with other insects [44]. One haplotype of *L. zonatus* has a wide distribution through much of this species’ known range, and the full distribution of the other lineage is unknown. To date, the five haplotypes uncovered are a relatively small number, and a higher number is expected in the insect’s center of origin. The introductions into Louisiana and Florida in the last few decades and the expansion into the northern Central Valley of California demonstrates the insect is expanding its range. Invasive species can be defined as those which are introduced, may transmit disease, cause economic damage, or outcompete native organisms [45]. While *L. zonatus* has become more abundant, expanded its range, and it can transmit plant pathogens, it is not known to kill trees directly. It does however cause economic damage in almonds, and is one of the most potentially damaging insect pests in almonds after navel orangeworm [13]. Recent decades have seen expansive planting of almonds in California which provides abundant food for these insects for much of the growing season. The negative effects of *L. zonatus* as a plant pest may outweigh the benefits of this insect in California, but would need to be determined [46]. There are many factors to consider, along with genetic diversity and an expanding range, to classify an invasive species.

## Figures and Tables

**Table 1:** *L. zonatus* samples which produced sequences in this study. *L. vexillatus* is a synonym of *L. zonatus. L. neovexillatus* was included as an outgroup. Mex = Mexico. Sample collection, coordinates, date, and *L. zonatus* strain. Samples are *L. zonatus* unless noted.

*L.z.* #	Collection Locality Country, State/City, Other	Latitude, Longitude	Collection Date	Strain
1	Mex, Queretaro-Santa Rosa Jauregui	20°42′22″ N, 100°26′34″ W	27 September 2006	2
2	Mex, Sinaloa, Concordia	23°19′47″ N, 105°58′40″ W	26 September 2010	2
3	Mex, Nayarit, Altavista, Compostela	21°4′27.08″ N, 105°8′56.49″ W *	5 June 2012	2
4	Mex, Yucatan, Yaxcopoil	20°44′26.20″ N, 89°43′22.07″ W *	5 August 2002	2
5	Mex, Colima	18°59′43.9″ N, 103°45′23.9′ W	25 November 2006	2
7	Bolivia, Chapare	16°44′6.38″ S, 65°37′0.29″ W *	31 March 2000	2
8	Mex, Nayarit, Ahuacatlan	20°59′ 06″ N, 104°27′08″ W	13 June 2009	2
11	Mex, Yucatan, Kinchil	20°54′39.96″ N, 89°56′59.15″ W *	16 August 2002	2
14	Mex, Guanajato, San Juan La Lagunita	21°34′22.56″ N, 101°32′2.71″ W *	9 November 2006	2
15	Mexico, Sonora	28°32′21.7″ N, 109°41′31.5″ W	15 September 2004	1
18	Mex, Chiapas, Tuxla Gutierrez	16°46′05.6″ N, 93°08′39.2″ W	3 July 2003	2
19	Mex, Oaxaca	17°3′41.30″ N, 96°43′17.08″ W *	15 July 2000	2
21	Mex, Oaxaca, El Charquito	17°5′2.16″ N, 96°40′5.41″ W *	19 June 1982	2
22	Mex, Guanajuato, San Juan La Lagunita	21°29′54.83″ N, 101°25′25.60″ W *	9 November 2006	2
28	Mex, Chiapas, Cintalapa Ejido Tehuacan	16°35′41.3″ N, 93°08′43.6″ W	25 June 2003	2
30	Mex, Oaxaca, Dominguillo	17°38′907″ N, 96°54′703″ W	18 October 1998	2
33	Nicaragua, Masaya	11°58′2.94″ N, 86°5′18.29″ W *	26 November 1991	2
48	*L. vexillatus*, Ecuador, Imbabura	0°20′22.98″ N, 78°7′42.09″ W *	12 December 1993	1
53	Mexico, Sonora, Tecoripa	28°37′19.5″ N, 109°57′0″ W	16 December 2004	2
54	Mex, Sinaloa, Concordia km32, VUD ^1^	23°19′47″ N, 105°58′40″ W	26 September 2010	2
55	Mex, Sinaloa, Concordia km32 VUD ^1^	23°19′47″ N, 105°58′40″ W	26 September 2010	2
56	Mex, Sinaloa, Concordia km72 VUD ^1^	23°27′29″ N, 105°49′51″ W	26 September 2010	2
57	Mex, Sinaloa, Concordia km32 VUD ^1^	23°19′47″ N, 105°58′40″ W	26 September 2010	1
60	Mex, Tamaulipas, Altamira km49 TCV	22°31′49″ N, 98°07′49″ W	9 May 2007	2
61	Mex, Tamaulipas, Gomez Farias, EEA ^1^	25°04′55″ N, 99°09′41″ W	12 May 2007	2
63	Mex, San Luis Potosí, 3 km N SMA ^1^	22°03′52″ N, 100°31′00″ W	20 August 2008	2
66	Mex, Guanajuato, San Juan Lagunita	21°33′38.37″ N, 101°28′55.63″ W *	9 November 2006	2
68	Mex, Guanajuato, San Juan Lagunita	21°33′49.97″ N, 101°34′53.30″ W *	9 November 2006	2
69	Mex, Queretaro, San Juan de Rio	20°22′02″ N, 100°01′13″ W	24 October 2007	2
70	Mex, Queretaro, Carretera La Venta-Lira	20°30′51.64″ N, 100°9′39.31″ W *	12 September 2007	2
71	Mex, Queretaro, Galindo, SMG ^1^	20°22′57.92″ N, 100°5′14.84″ W *	22 November 2011	1
73	Mex, Queretaro, Carretera La Venta-Lira	20°30′55.72″ N, 100°9′44.06″ W *	12 September 2007	1
75	Mex, Morelos, Tepalcingo, El Limon	18°32′34″ N, 98°56′104″ W	23 October 2006	2
	***L. neovexillatus*** *			
43	Bolivia, Andres Ibanez, Canton Terebinto	17°46′15.46″ S, 63°21′40.52″ W *	18 March 2005	n/a
44	Bolivia, Santa Cruz, Potrerillo de Guenda	17°45′16.39″ S, 63°14′53.63″ W *	13 October 2011	n/a

^1^ VUD = Villa Union Durango; TCV = Tampico Cuidad Victoria, SMA = Santa Maria de Abajo; EEA = Ejido El Azteca; SMG= San Miguel Galindo. * indicates a latitude/longitude was estimated. * Two *L. neovexillatus* were included.

**Table 2:** Collection year and sequencing outcome.

Year Collected	Sequence Obtained	Not Obtained
2000–2012	31	13
Before 1999	4	27

**Table 3:** The number of haplotypes, haplotype diversity, and nucleotide diversity for populations of *L. zonatus*.

Lz Collection (n)	Number of Haplotypes	Haplotype Diversity	Nucleotide Diversity
California (41)	3	0.526	0.008
Cal. lineage 1 (24)	2	0.083	0.008
Cal. lineage 2 (17)	1	0.000	0.000
Mexico (30)	3	0.248	0.002
Mex. lineage 1 (4)	2	0.667	0.002
Mex. lineage 2 (26)	1	0.000	0.000
Brazil (37) (all lineage 2)	3	0.505	0.001
Combined lineage 1 and 2 (111)	5	0.538	0.006
Lz lineage 1 Cal./Mex. (28)	2	0.198	0.001
Lz lineage 2 Cal./Mex. (43)	1	0.000	0.000
Lz lineage 2 All (82) *	3	0.245	0.001

* Combined data set includes 3 samples from Nicaragua, Bolivia, and Ecuador. See Figure 1 phylogeny for strain 1 and strain 2 samples, and see map Figure 2. Ca = California, Mx = Mexico, Lz-*L. zonatus.*

## Data Availability

Sequences used in this study have the following Genbank Accession numbers: *Leptoglossus zonatus* OL757664-96, *Leptoglossus neovexillatus* OL764685-86.

## Supplementary Materials

The following are available online at https://www.mdpi.com/article/10.3390/insects12121094/s1, Table S1: *L. zonatus* museum samples which did not produce a DNA sequence. Mex = Mexico. *L. vexillatus = L. zonatus*.
