# Supplementary material for: Distribution of Two Strains of Leptoglossus zonatus (Dallas) (Hemiptera: Coreidae) in the Western Hemisphere: Is L. zonatus a Potential Invasive Species in California?
Source: Insects. 2021 Dec 7;12(12):1094. doi: 10.3390/insects12121094 (PMC8703490; doi:10.3390/insects12121094)
Supplement: Supplementary file 1 [file insects-12-01094-s001.zip › insects-1479643-supplementary.pdf]

Supplemental Table S1. *L. zonatus* museum samples which did not produce a DNA sequence. Mex=Mexico. *L. vexillatus*=*L. zonatus*.

Number	Collection Locality	Collection Date
6	Venezuela, Aragua, Maracay	Aug. 29, 1975
9	Bolivia, Santa Cruz	April 24, 2005
10	*L.vexillatus* (=*L. zonatus*), Bolivia, Santa Cruz, B. vista	Mar 14, 1957
12	Mex, Yucatan, Yaxcopoil	Sept 13, 2002
13	Mex,Oaxaca	Oct. 26, 2007
16	Mex, Veracruz, San Andres Tuxla	May 11, 1972
20	Mex, Veracruz, Xalapa	Sept 1985
23	Mex, Tamaulipas,28 mile NE Tula	July,22, 1982
24	Mex,Aguascalientes	April 11, 2002
25	Mex, Puebla, 6 km No Teontepec	July 14, 1999
26	Mex, Jalisco, Chapala	Sep. 18, 1995
27	Peru	1967
29	Mex, Queretaro	July 21, 1998
31	Honduras, Morazon, Zamorano School	July 13, 1948
32	Honduras El Paraiso, Rapaco	Oct. 8, 1986
34	Panama	*
35	Guatemala Escuintla	June 30, 1987
36	Honduras El Paraiso, Rapaco	Aug. 22, 1986
37	Venezuela, Aragua, Maracay	Aug.29, 1975
38	Colombia, Tolima	*
39	Colombia, Medellin	Aug. 1983
40	Venezuela, Aragua, El Limon	June 5, 1976
41	Venezuela, Aragua	June 29 1974
42	Colombia, Tolima	*
45	Peru, Huanuco, Tingo Maria	*
46	Peru, Pilcopata	Feb 6, 1975
47	*L. vexillatus*, Ecuador, Pichincha Puerto Quito	Oct. 1983
49	*L. vexillatus* , Pichincha Cumbaya	April 17, 1992
50	*L. vexillatus*, Ecuador Prov Manabi Montecristo	Feb. 1983
51	*L. vexillatus* Ecuador Pichin Tumbaco	June 23, 1984
52	Mexico, Sonora, Municipio de Alamos	July 8, 2012
58	Mex,Tamaulipas, Altamira km49 Tampico-Cuidad Victoria	May 9, 2007
59	Mex,Tamaulipas,Altamira km49 Tampico-Cuidad Victoria	May 9, 2007
62	Mex,Tamaulipas,Altamira km49 Tampico-Cuidad Victoria	May 9, 2007
64	Mex, San Luis Potosí, 3 km N of Santa Maria de Abajo	Aug. 20, 2008
65	Mex, Guanajuato, San Juan Lagunita, Km 18 Carretera Queretaro	Nov 9, 2006
67	Mex, Aguascalientes,Salto de Los Salado el Sabinal	April 11, 2002
72	Mex, Queretaro, Carretera la Venta-Lira	Sept. 12, 2007
74	Mex,Morelos,Tepalcingo,CEAMISH	Aug. 30, 2004

* Indicates a specimen missing the collection date
